# 1619. Epidemiology of Congenital Syphilis Infections in Northwestern (NW) Iowa Witnessing a Significant Increase- Experience of a Tertiary Care Center

**DOI:** 10.1093/ofid/ofad500.1454

**Published:** 2023-11-27

**Authors:** Ashlesha Kaushik, Kristen Beal, Sandeep Gupta

**Affiliations:** UnityPoint Health and University of Iowa Carver College of Medicine, Sioux City, Iowa; UnityPoint Health, Sioux City, Iowa; Pulmonary and Critical Care Medicine, Unity Point Health, Sioux City, Iowa

## Abstract

**Background:**

According to the CDC, STI (sexually transmitted infections) including syphilis infections in adults have recently increased in the upper Midwest. Studies evaluating epidemiology of Congenital syphilis (CS) are needed to facilitate prompt recognition to prevent sequelae.

**Methods:**

We retrospectively studied epidemiologic, clinical characteristics and outcomes of neonates with CS at our tertiary care center which is the largest maternal/neonatal center in NW Iowa during 1/1/2021-12/31/2022 (labeled P2). Epidemiologic characteristics during P2 were compared with the period from 1/1/2019-12/31/2020 (labeled P1). Diagnosis and treatment for CS were in accordance with CDC/American Academy of Pediatrics Committee on Infectious Diseases Red Book guidelines.

**Results:**

During P2, a total of 42 newborns were diagnosed with CS compared to 0 during P1 (p< 0.05); 64% of infected newborns were White followed by Native American (21%), Hispanic (11%) and Black (2%). All (100%) neonates with CS were born to mothers infected during or after second trimester of pregnancy with negative first trimester RPR (Rapid Plasma Reagin) testing. Maternal RPR titers ranged from 1:1-1:256. Majority (62%) of newborns were diagnosed with proven/possible CS with RPR-titers > 4-fold mother's titers /untreated or inadequately treated maternal infection and required 10 days of intravenous Aqueous crystalline penicillin G treatment and NICU care; 2 newborns had meningitis and 3 developed sensorineural hearing loss. Thirty-eight% received 1 dose of intramuscular Benzathine penicillin G. Need for cerebrospinal fluid syphilis testing and 10 days of intravenous penicillin treatment increased by > 600% from P1 to P2 (p=0.02). CS evaluation/treatment in infected Native American neonates increased by > 1000% in P2 (p< 0.001). Mean hospitalization duration was 9.5 (±1.6) days. No infant died. Only 33% of infants followed up at 2 months for repeat testing. None of the Native American/Hispanic/Black neonates compared to 52% White neonates had follow-up testing (p< 0.05).

**Conclusion:**

Our results show an alarming increase in CS in NW Iowa and underline the need for urgent preventive measures including STI prevention education, adequate maternal/ neonatal testing and follow-up, especially among marginalized populations.

**Disclosures:**

**All Authors**: No reported disclosures

